# Outcomes analysis of new entrant screening for active tuberculosis in Heathrow and Gatwick airports, United Kingdom 2009/2010

**DOI:** 10.1186/s12879-016-1506-2

**Published:** 2016-04-22

**Authors:** Ettore Severi, Helen Maguire, Chikwe Ihekweazu, Graham Bickler, Ibrahim Abubakar

**Affiliations:** European Programme for Intervention Epidemiology Training (EPIET), European Centre for Disease Prevention and Control, Stockholm, 17183 Sweden; Health Protection Agency (HPA), London, SW1W 9SZ UK; Present address: European Centre for Disease Prevention and Control, Surveillance and Response Support Unit, Stockholm, 17183 Sweden; University College London, Centre for Infectious Disease Epidemiology, London, WC1E 6BT UK; Health Protection Agency, Colindale, NW9 5EQ UK

**Keywords:** Communicable diseases, Immigration, Respiratory tract diseases, Diagnostic accuracy, Tuberculosis

## Abstract

**Background:**

In 2012, the United Kingdom (UK) Government announced that the new entrant screening for active tuberculosis (TB) in Heathrow and Gatwick airports would end. Our study objective was to estimate screening yield and diagnostic accuracy, and identify those at risk of active TB after entry.

**Methods:**

We designed a retrospective cohort study and linked new entrants screened from June 2009 to September 2010 through probabilistic matching with UK Enhanced TB Surveillance (ETS) data (June 2009 to December 2010). Yield was the proportion of cases reported to ETS within three months of airport screening in the screened population. To estimate screening diagnostic accuracy we assessed sensitivity, specificity, positive and negative predictive values. Through Poisson regression we identified groups at increased risk of TB diagnosis after entry.

**Results:**

We identified 200,199 screened entrants, of these 59 had suspected TB at screening and were reported within 3 months to ETS (yield = 0.03 %). Sensitivity was 26 %; specificity was 99.7 %; positive predictive value was 13.2 %; negative predictive value was 99.9 %. Overall, 350 entrants were reported in ETS. Persons from countries with annual TB incidence higher than 150 cases per 100,000 population and refugees and asylum seekers were at increased risk of TB diagnosis after entry (population attributable risk 77 and 3 % respectively).

**Conclusion:**

Airport screening has very low screening yields, sensitivity and positive predictive value. New entrants coming from countries with annual TB incidence higher than 150 per 100,000 population, refugees and asylum seekers should be prioritised at pre- or post-entry screening.

## Background

The incidence of tuberculosis (TB) in the United Kingdom (UK) increased between 1990 and 2005, stabilized until 2011 and decreased in the three following years. In 2014, 6622 cases of TB were reported in England – an incidence of 12.0 cases per 100,000 population [1–3]. In 2010, 73 % of newly diagnosed TB cases with a known country of birth were born outside the UK; TB incidence among these was 81.6 per 100,000 population, around 20 times higher than those born in the UK (3.9 per 100,000 population). Most of these cases were born in South Asia (55 %) or Sub-Saharan Africa (26 %); regions with the highest incidence of TB disease in the world [4]. The majority of cases born out of the UK (77 %) were diagnosed two or more years after entering the UK [5].

In response to increasing migration to the UK of individuals born in TB high risk countries, the UK government set up new entrant screening for active TB at ports of entry (“new entrants screening”) in 1965 [6]. Chest x-rays were introduced at London Heathrow airport for long-stay new entrants. Screening was subsequently extended to London Gatwick airport. The aim of screening was to reduce importation of TB into the UK at entry by identifying active pulmonary disease among those who were seeking to remain in the UK for longer than six months so as to prevent onward transmission [7]. Those making shorter visits to the UK were not routinely screened for TB.

Different studies on cohorts of new entrants and reviews of screening practices have concluded that the new entrants screening programme in the UK was an ineffective and inefficient method for detecting active cases of TB [8–12]. While they provided a consistent evaluation over a long period of time, their findings were limited, they were only undertaken at local level [8, 9, 11, 12] or did not have a robust study design by not taking into account TB cases subsequently diagnosed after entry [10].

The aim of our large population based study, undertaken in March 2011, was to review the yield and the outcomes of new entrant screening at Heathrow and Gatwick to contribute to the advice of the Health Protection Agency (HPA) to the UK government on the future of on-entry screening.

In May 2012, there was a UK government announcement that on entry screening would end. It was discontinued in summer 2012 at Gatwick airport and on 31 March 2014 at Heathrow airport [13]. Our study objectives were: i) to estimate the yield of the screening: i.e. the proportion of true TB cases among all screened new entrants; ii) to estimate the screening diagnostic accuracy in terms of sensitivity, specificity, positive predictive value (PPV) and negative predictive value (NPV) related to the suspicion of active TB at screening; iii) to identify the risk factors associated with an increased risk of TB diagnosis after entering the UK.

## Methods

According to the UK government protocol guiding the new entrants screening policy, individuals entering the UK older than 15 years of age, not pregnant, subject to immigration control, from a country with an estimated TB incidence greater than 40 per 100,000 (UK definition of high incidence country[14]) and intending to remain in the UK for longer than 6 months were referred by UK Borders Agency staff to the airports’ Health Control Units (HCU) for screening [7, 10]. New entrants referred to the HCU underwent a medical assessment for TB, which could include a chest x-ray. Those suspected of having active TB were invited to arrange urgent follow up and register with the medical services close to their intended residence. The HCU also sent notification of the new entrants suspected of active TB to the National Health System (NHS) including the address of the entrant for follow up. All new entrants not suspected of having active TB at the time of entry were also invited to register with a general practitioner for follow up, mainly for checking BCG vaccination status and possibly arranging for latent TB screening if relevant.

To achieve the study objectives we designed a retrospective cohort study including all new entrants screened at Heathrow and Gatwick airports from 10 June 2009 to 30 September 2010. Information collected for new entrants included demographic (name, date of birth and sex), date and visa of entry, nationality and address in the UK declared at entry. Identification of those new entrants who had a TB diagnosis at any point after entry was determined by matching the cohort of new entrants with notifications of TB diagnosis from the Enhanced Tuberculosis Surveillance database (ETS) from 1 June 2009 to 31 December 2010. ETS is the surveillance system reporting active TB cases notified by TB clinics in the UK [15]. We matched the two datasets using a probabilistic algorithm on the basis of available personal identifiers (name, surname, sex, date of birth, nationality, address) [16].

We defined: i) a TB suspect as any new entrant suspected of active TB at screening; ii) a TB diagnosis as any new entrant who was later notified in ETS with TB; iii) a TB diagnosis attributable to screening as any new entrant suspected of TB at screening, later notified in ETS within 90 days from screening.

We defined the yield of TB attributable to the screening as the proportion of new entrants suspected and diagnosed with TB within 90 days after screening among all screened new entrants during the study period. We defined 90 days as the necessary maximum time for a new entrant to follow the indication received at screening, including the time allowed to come into contact with the NHS.

In order to assess the screening diagnostic accuracy related to the suspicion of active TB at screening, we used ETS as the gold standard. We calculated sensitivity as the proportion of new entrants suspected of TB among those diagnosed with TB; specificity as the proportion of new entrants not suspected of TB among those not diagnosed with TB; PPV as the proportion of new entrants diagnosed with TB among those suspected of TB; NPV as the proportion of new entrants not diagnosed with TB among those not suspected of TB.

Finally, we used Poisson regression to identify the groups of new entrants at increased risk of TB diagnosis after screening. We considered as cases all individuals diagnosed with TB at any time within the follow up period after entry to UK. We took into account age, sex, type of visa they had been issued and TB incidence in the country of origin. We categorised study participants into: i) four age groups (16–24 years, 25–29 years, 30–54 years and 55 years or older); ii) male and female sex; iii) four visa categories (student, work permit, long stay visitor and refugee or asylum seekers); iv) country of origin, grouping countries on the basis of their TB incidence in four groups (TB incidence 40–150, including China, Russia and several central and south American countries; TB incidence 151–250, including India, Pakistan, Indonesia and Bangladesh; TB incidence 251–350, including Nigeria and several sub-Saharan countries; TB incidence >350, including South Africa and several other sub-Saharan countries). Participants’ missing information were excluded from the analysis. Subsequently identifying the groups at increased risk of TB diagnosis after screening, we also estimated their population attributable risk (PAR), calculating the proportion of TB cases explained by these groups among the overall number of TB cases detected in our cohort.

## Results

We had a total of 200,199 new entrants in our cohort, 57 % were male and over 75 % were younger than 30 years old (median age 25 years, IQR 22–29 years). New entrants into the UK with student visa were 69.2 %. Most new entrants were coming from countries with a TB incidence between 150 and 250 per 100,000 population (Table 1).

**Table 1:** Short title: main characteristics of new entrants screened in Heathrow and Gatwick, UK, 2009/2010. Detailed title: distribution of new entrants on the basis of demographic characteristics, visa of entry and tuberculosis (TB) incidence in the country of origin

	No.	(%)
Sex		
Male	114 191	(57.0)
Age (years)		
16–24	87 222	(43.6)
25–29	68 762	(34.4)
30–54	41 501	(20.7)
≥55	2 678	(1.3)
Unknown	36	(0.0)
Visa of entry		
Student	138 466	(69.2)
Work permit	32 006	(16.0)
Long Stay Visitor	6 321	(3.2)
Refugee/Asylum Seeker	1 913	(1.0)
Unknown	21 493	(10.7)
Incidence of TB in country of origin (annual cases per 100 000)		
40–149	57 541	(28.7)
150–249	106 686	(53.3)
250–349	22 001	(11.0)
≥350	5 288	(2.6)
Unknown	8 683	(4.3)
Total	200 199	(100.0)

**Fig. 1:**
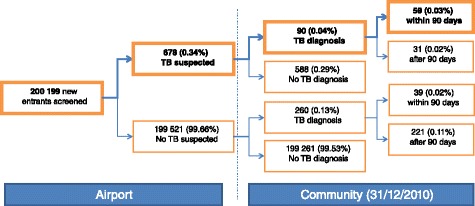
Short title: Distribution of new entrants on tuberculosis suspicion at entry and tuberculosis diagnosis at community level. Detailed legend: Distribution of new entrants screened in Heathrow and Gatwick airports on the basis of suspicion of tuberculosis at entry screening in the United Kingdom and tuberculosis diagnosis at community level through identification in the Enhanced TB Surveillance System (ETS)

Figure 1 shows the distribution of new entrants based on the suspicion of TB at entry and on subsequent TB diagnosis (those identified in ETS). Of the 200,199 new entrants screened, 678 (0.34 %) were suspected of having TB at entry. Of these 90 (0.04 %) were later notified in ETS with TB, of which 59 were diagnosed within 90 days after screening. Therefore 59 entrants, representing 0.03 % of the total cohort screened, had a TB diagnosis we attributed to the screening process.

The screening test sensitivity (Table 2) was 25.7 % (90/350); specificity was 99.7 % (199 261/199849); PPV was 13.3 % (90/678) and NPV 99.9 % (199261/199521).

**Table 2:** Short title: distribution of new entrants on tuberculosis suspicion at entry and diagnosis at community level. Detailed title: distribution of new entrants on the basis of active tuberculosis (TB) suspicion identification at entry screening and tuberculosis diagnosis at community level (identified in the Enhanced TB Surveillance System (ETS))

		TB diagnosis in ETS		
		Yes	No	Total
Suspiscion at entry	Yes	90	588	678
	No	260	199 261	199 521
Total		350	199 849	200 199

A total of 350 new entrants (0.17 %) were diagnosed with TB at any time within the follow up period after entry to the UK (Table 3). Taking into account sex, age, type of visa and country of provenance, we did not find any association between sex or age and TB diagnosis. Refugees and asylum seekers were found to have about 4 times increased risk (adjusted risk ratio ARR 4.27, *p* <0.01) of TB diagnosis after entry when compared to students. Any new entrant from a country with a TB incidence ≥150 was approximately 5 to 6 times more at risk of TB diagnosis than those coming from countries with a TB incidence between 40 and 150 (ARR > 4.93; *p* <0.01). Finally, in our cohort the PAR of entering into the UK as an asylum seeker or a refugee was 0.03 and the PAR of coming from a country with a TB incidence ≥150 was 0.77; thus respectively three per cent and 77 % of the cases in our cohort could be explained by these characteristics.

**Table 3:** Short title: characteristics of new entrants with tuberculosis and risk factors for tuberculosis diagnosis. Detailed title: characteristics of new entrants diagnosed with tuberculosis (TB) at any time after entry screening and adjusted risk ratio (ARR), 95 % confidence interval (95 % CI) and *P* value for the association between exposure category and TB diagnosis at community level (identified in the Enhanced TB Surveillance System (ETS))

	TB cases (no. = 350)	TB cases (%)	Adjusted RR	95 % CI		*P* value (Wald test)
Sex						
Female	149	(0.17)	Ref.			
Male	201	(0.18)	0.91	0.70	1.18	0.48
Age (years)						
16–24	117	(0.13)	Ref.			
25–29	134	(0.19)	1.15	0.87	1.52	0.34
30–54	83	(0.20)	1.05	0.73	1.49	0.81
≥55	16	(0.60)	2.36	0.56	9.92	0.24
Visa of entry						
Student	197	(0.14)	Ref.			
Work permit	52	(0.16)	0.78	0.55	1.10	0.16
Long stay visitor	9	(0.14)	0.81	0.40	1.65	0.56
Refugee/Asylum Seeker	10	(0.52)	4.27	2.24	8.13	<0.01
Incidence of TB in country of origin (cases per 100,000 population per year)						
40–150	25	(0.04)	Ref.			
151–250	241	(0.23)	6.14	3.77	10.00	<0.01
251–350	55	(0.25)	4.93	2.40	7.61	<0.01
>350	19	(0.36)	5.53	2.14	14.33	<0.01

## Discussion

In a large study of over 200,000 new entrants to the UK we found a very low yield of TB, with only 59 TB diagnoses attributable to the screening in a time period of 15 months. In order to put this figure in context, about 8500 new cases of TB were notified in 2010 in the UK and 80 % of these were born out of the UK [5]. In addition, chest X-rays are able to detect mainly pulmonary TB; in 2010, 54 % of the TB diagnoses reported in individuals born out of the UK were extra-pulmonary [5]. This is a serious limitation as chest X-rays will not detect extra-pulmonary TB. Furthermore, the new entrants screening was performed only in Heathrow and Gatwick airports and in no other UK port of entries. In 2009 and 2010, likewise in 2016, only a proportion of new entrants to the UK entered through these two airports; in fact a growing proportion of new entrants to the UK enter through other international airports, maritime ports and railways connecting the UK with continental Europe. In a 2009 systematic review of the different new entrant TB screenings in the European Economic Area and Switzerland, Klinkenberg et al. identified three different screening strategies: at port of entry, just after arrival in reception centres and post-entry at community level. The median yield of these strategies was about 10 times higher than the UK’s new entrant screening; screening at reception centres had the highest yields [17]. In the same review, yields for screening programs in reception centres and post-entry at community level in Australia, Canada, Japan and U.S.A. were also assessed: the highest median yield, 40 times higher than the UK’s new entrant screening, was obtained in pre-entry screening. No information was available on screening at entry ports for these countries [17]. Zenner et al. in a 2013 primary cost effectiveness study identified comparable yields among each screening strategy with at entry screening still not found to be cost-effective and having little impact on overall TB trends [18]. In 2009 and 2014 in the USA, Liu et al. and Posey et al., evaluating pre-entry (overseas) screening, identified yields substantially higher than in the UK new entrants screening [19, 20].

The diagnostic accuracy analysis showed screening had very poor sensitivity. On the other hand, specificity was nearly 100 %. Due to the very high prevalence of new entrants without active TB at entry PPV was very low and NPV almost 100 %.

When we looked at the groups of new entrants at increased risk of TB diagnosis at any follow up point after screening, we found refugees and asylum seekers having the highest risk compared to the other VISA categories of entrants. In this analysis, students were selected as the comparison group because they were the most numerous group and those with the lowest risk of TB. When compared with any other visa category, refugees and asylum seekers were always found to be at increased risk. Due to the low number of refugees or asylum seekers entering in the UK in 2009 and 2010, only three per cent of the cases in our cohort were in this group. However, these findings are of increasing interest as the proportion of refugees and asylum seekers has steeply grown in recent years in the UK comprising an estimated 8 % of net migration in 2013, comparing with an estimated 4 % in 2010 [21]. The number of refugees and asylum seekers to the UK has further increased in 2015. This finding is even more important at European Union level, where first time applicant asylum seekers reached the number of 1,255,640 in 2015 [22].

We also found that new entrants from a country with a TB incidence higher than 150 per 100,000 population were at an increased risk of TB diagnosis when compared with new entrants from countries with a TB incidence lower than 150 per 100,000. The analysis of the PAR for this group highlighted that more than 75 % of the cases in our cohort came from a country with an incidence ≥150 per 100,000. In these analyses we used Poisson regression to estimate the risk. Using logistic regression we obtained very similar estimate of odds (data not shown). A number of recent studies found that screening for latent TB among new entrants from countries with a TB incidence greater than 150 per 100,000 is the most cost-effective strategy to identify latent TB [23–25]. This threshold makes an important difference, since those countries with a TB incidence between 150 and 250 per 100,000 include India, the country contributing the largest number of new entrants into the UK. Consistent with this, the 2011 guidelines of the National Institutes for Health and Clinical Excellence (NICE) for TB diagnosis and management recommended offering treatment for latent TB to *any* new entrant from a country with a TB incidence greater than 40 per 100,000, rather than the previous policy of offering TB treatment only to those new entrants from a country with TB incidence greater than 500 per 100,000, which included mostly sub-Saharan Africa [26].

Our study results are based on a robust study design and on a very large sample of individuals. On the other hand, our study had a number of limitations. The probabilistic matching between the new entrant database and ETS could have resulted in incomplete or over- matching. However, the accuracy of our probabilistic matching system is considered high [16], thus we expect the proportion of mismatched individuals to be very low and this would underestimate any association identified in the study. At the time of the matching we had information only from the TB surveillance systems in England, Wales and Northern Ireland. Data from Scotland were not yet available. Therefore we may have missed a few new entrants who moved to Scotland and were reported with TB by the Scottish health system. Since the proportion of TB cases born out of the UK in Scotland in 2012 was 57 % compared to 74 % in the rest of the United Kingdom [14], we expect these to be very few individuals. However, this could have slightly under-estimated the true screening yield.

Some new entrants may have left the UK before being diagnosed or may have been missed by ETS. This could also result in an underestimation of the true screening yield. Although not perfect, we used ETS as gold standard in the diagnostic accuracy analysis. Ideally we should have traced and tested new entrants after screening; however, due to the very large number of new entrants screened, this was not practical. Finally, depending on the time of arrival, and since the ETS data were available only until the end of 2010, new entrants had a different probability of being notified in ETS. This was due to the availability of ETS data when the study was performed and may have resulted in some TB notifications in ETS being missed. Therefore the study could have slightly under-estimated the true screening yield.

The epidemiology of TB in the UK is mainly driven by reactivation of latent TB acquired before migration [27, 28]. In 2010, over 75 % TB cases born out of the UK (77 %) were diagnosed two or more years after entering the UK; therefore, we would expect several new entrants to be diagnosed with TB in the five years following the end of our study. However, we do not think that the profile of those diagnosed will change and we expect the relative risk associated with asylum seeking and refugee status not to change appreciably.

## Conclusions

The study provides critical evidence to support the HPA’s advice and the UK government decision to discontinue new entrant screening for active tuberculosis at Heathrow and Gatwick airports and to opt for other screening strategies.

New entrants coming from countries with annual TB incidence over 150 per 100,000 populations, refugees and asylum seekers should be urgently linked to health services after arrival in the UK and offered a test for latent TB; they should also be prioritised in any future screening. Similar public health actions should also be implemented in all countries recently affected by the large movement of people into the European Union [22].

It is also important to repeat the analysis of which groups in our cohort are at increased risk of TB diagnosis after entry with 5 years of follow up to make sure that the profile of those reported after entry has not changed.

Finally, screening strategies should be regularly evaluated to make sure that the screening expectations are met and the screening is operating in the best public health interest.

### Ethics approval and consent to participate

This is a secondary data analysis of information collected for the purpose of the UK Port of Entry Screening. All data are presented in aggregated format. No individual participants’ data is presented in any form.

### Consent for publication

Not applicable.

### Availability of data and materials

Data will not be shared. Data ownership is not up to the authors of this publication and no permission for sharing has been granted to them.

## Abbreviations

ARR: adjusted risk ratio
ETS: Enhanced Tuberculosis Surveillance
HCU: Health Control Units
HPA: Health Protection Agency
NHS: National Health System
NICE: National Institutes for Health and Clinical Excellence
NPV: negative predictive value
PAR: population attributable risk
PPV: positive predictive value
TB: Tuberculosis
UK: United Kingdom
